# Implementing a protocol for a research impact assessment of the Centre for Research Excellence in Stroke Rehabilitation and Brain Recovery

**DOI:** 10.1186/s12961-018-0349-2

**Published:** 2018-08-01

**Authors:** Shanthi Ramanathan, Penny Reeves, Simon Deeming, Julie Bernhardt, Michael Nilsson, Dominique A. Cadilhac, Frederick Rohan Walker, Leeanne Carey, Sandy Middleton, Elizabeth Lynch, Andrew Searles

**Affiliations:** 1 Health Research Economics, Hunter Medical Research Insitute, Locked Bag 1, New Lambton Heights, NSW 2305 Australia; 2 School of Medicine and Public Health, Faculty of Health and Medicine, University of Newcastle, Callaghan, NSW Australia; 3 The Florey Institute of Neuroscience and Mental Health, Melbourne University, Melbourne, VIC Australia; 4 NHMRC Centre of Research Excellence in Stroke Rehabilitation and Brain Recovery, Heidelberg, VIC Australia; 5 Stroke and Ageing Research, School of Clinical Sciences at Monash Health, Monash University, Clayton, VIC Australia; 6 School of Allied Health, College of Science, Health and Engineering, Department of Community and Clinical Allied Health, LaTrobe University, Bundoora, VIC Australia; 7 Nursing Research Institute, St Vincent’s Health Australia (Sydney) and Australian Catholic University, Darlinghurst, NSW Australia; 8 Adelaide Nursing School, Faculty of Health and Medical Sciences, University of Adelaide, North Terrace, SA Australia

**Keywords:** Impact assessment, Research translation, Stroke rehabilitation, Health economics

## Abstract

**Background:**

There is growing recognition that the wider benefits of research (economic, social and health impacts) should be assessed and valued alongside traditional research performance metrics such as peer-reviewed papers. Translation of findings into policy and practice needs to accelerate and pathways to impact need to be better understood. This research protocol outlines a mixed methods study to apply the Framework to Assess the Impact from Translational health research (FAIT) to the Centre for Research Excellence in Stroke Rehabilitation and Brain Recovery (CRE-Stroke). FAIT is purpose-designed to encourage research translation and assess research impact but lacks validation.

**Methods/Design:**

Phase 1 involves application of the FAIT-modified programme logic model to each CRE-Stroke research stream including identifying process, output and impact metrics, as well as end users of the research. A scoping review will inform potential impacts anticipated from CRE-Stroke. In Phase 2, audit and feedback on achievements against plans will track and encourage research translation. Logic models will be updated to account for changes in the research pathways over time. In Phase 3, three proven methods for measuring research impact – Payback, economic assessment and narratives – will be applied to each research stream and the data triangulated and reported in Phase 4. The feasibility of applying FAIT will also be assessed as part of Phase 3.

**Discussion:**

Use of prospective, comprehensive research impact frameworks for large interdisciplinary programmes of research is rare. FAIT’s application to CRE-Stroke will provide opportunity for the impact of CRE-Stroke to be assessed and a range of impacts beyond standard academic achievements to be reliably reported. The feasibility of FAIT’s application will also be assessed and, if necessary, refined. The usefulness of FAIT for encouraging research translation will also be described and may prove useful for other programmes looking to implement a research impact framework.

## Background

Stroke is a leading cause of death and disability around the world [1]. In Australia, stroke is the leading cause of disability, with over 50,000 stroke cases annually, and the second largest cause of death [2]. In 2012, there were over 420,000 stroke survivors in Australia living with the effects of stroke [3]. The total financial cost of stroke in Australia was estimated to be close to AUD 5 billion in 2012. However, the biggest impact of stroke is the loss of healthy life. Using the market-based price of risk methodology required by the Commonwealth Department of Finance and Deregulation, the total burden of disease cost for stroke in 2012 was AUD 49.3 billion [4].

In recent decades, there have been important advances in the field of stroke with the emergence of strong evidence for approaches to stroke recovery [5]. Nevertheless, there have been significant delays in implementing evidence into clinical practice [6, 7] and stroke survivors often do not receive care based on the best available evidence. For example, in Australia, one in five stroke survivors are still discharged without a care plan and only half are assessed for mental wellbeing [8, 9].

Recognising a need to expand the evidence base for rehabilitation interventions, improve recovery from stroke and reduce the burden of disease cost for stroke, the National Health and Medical Research Council (NHMRC) of Australia funded the Centre for Research Excellence in Stroke Rehabilitation and Brain Recovery (CRE-Stroke). The vision for CRE-Stroke is to transform the stroke rehabilitation research and practice landscape in Australia and accelerate the development, translation and implementation of new interventions that are strongly supported by neuroscience [10].

The NHMRC Centre for Research Excellence scheme provides support for teams of researchers to pursue collaborative research and develop capacity in clinical, population health or health services research. A major objective of the CRE-Stroke research programme is to use an impact framework to encourage research translation and assess the impact of its five research streams (Basic Science, Clinical Trials, Neuroimaging, Implementation and Data Linkage). This is because a substantial amount of health and medical research fails to be fully translated and is therefore not taken advantage of by end users for policy and practice, thus limiting impact [11]. There is also a growing demand for more accountability in public spending across all sectors, including health [12]. Any level of suboptimal translation means the potential rates of return from research investments may not be realised. In this protocol, we define ‘research translation’ as “*the process of knowledge generation and transfer that enables those utilising the developed knowledge to implement it*” [13]. The definition for ‘research impact’ modified for the health and medical research context is “*the demonstrable effect from basic, health systems, patient and population-orientated research, and clinical trials, that ultimately improves healthcare delivery, human health and quality of life, and generates benefits for the economy, society, culture, public policy, or the environment*” [14].

There is growing recognition that translation of research into policy and practice needs to increase and that the pathways to realising impact need to be more transparent [15]. Moreover, impact assessment beyond academic outputs such as peer-reviewed publication citations is still not standard practice in many countries [16]. One exception is the United Kingdom, which is leading the way in implementing an impact assessment agenda and, for the first time in 2014, included impact assessment in the national Research Excellence Framework [17]. There is a plethora of impact assessment frameworks available, including two recent systematic reviews of these frameworks, models and applications [18–20]. However, there is a lack of evidence to suggest that the availability of these frameworks and models has increased the proportion of health and medical research projects that assess and report on broad concepts of impact beyond the narrow scope of academic outputs.

There have been several studies trialling the use of impact assessment applications in Australia, but a national framework for assessment of research impact has not yet been implemented. However, there have been major national developments in this field, including (1) the Excellence in Research for Australia National framework [21], (2) NHMRC’s Advanced Health Research and Translation Centres Program and Centres for Innovation in Regional Health [22], (3) the Medical Research Future Fund [23], and (4) Australian Research Council’s national engagement and impact assessment framework [24, 25]. These initiatives confirm that research translation and impact assessment are currently high on Australia’s research agenda. Attempts to close the gap between research outputs and impacts are strongly encouraged and likely to remain policy relevant.

Recently, the Australian Commission on Safety and Quality in Healthcare released a report quantifying the overall health and economic impact of 25 investigator-initiated clinical trials conducted by three select clinical trials networks, including the Australasian Stroke Trials Network [26]. The report was able to demonstrate that increasing implementation of trial evidence into practice can lead to considerable additional health and economic gains. It showed that the results of these 25 trials only needed to be implemented in 11% of eligible populations for benefits to outweigh costs. This is one of the first reports in Australia to quantify impact from clinical trials and further similar studies are warranted.

The impact assessment framework selected to guide the review of CRE-Stroke is the Framework to Assess the Impact from Translational health research (FAIT), developed by a team of health economists and health and medical researchers based at the Hunter Medical Research Institute with the specific aim of encouraging and measuring research translation and impact. Details of the framework have been previously published and provide a comprehensive account of the development of FAIT and the components of the framework [27]. Briefly, the framework was based on a mixed methods study involving (1) a scoping review of existing research impact frameworks and techniques to inform the development of FAIT, (2) a development stage to design the prototype and (3) a feedback stage where iterations of the prototype were presented to researchers for discussion and refinement. FAIT is based on a modified programme logic model and a hybrid of three proven methodologies for measuring research impact; namely quantified metrics, economic analysis and narratives of the process by which research translates and generates impact. The adoption of FAIT by CRE-Stroke presents an opportunity to pilot the framework’s application and test the feasibility of its research translation capability. It also allows its impact assessment methodology to be applied to ‘streams of research’ rather than specific research projects. This paper describes the protocol of a mixed methods study to document the pathway to translation and assess the impact of five streams of stroke rehabilitation research associated with CRE-Stroke.

## Methods/design

This study involves the application of a research impact framework (FAIT) to encourage research translation and assess research impact [27]. The aims are to:

1. Provide transparency to the pathway to generating research impact
2. Test the utilisation of impact assessment to encourage greater research translation
3. Assess the impact of the five research streams of CRE-Stroke
4. Test the feasibility of using FAIT’s package of validated impact assessment methodologies on an interdisciplinary research programme in stroke
5. Build the knowledge and capability within the CRE-Stroke research team regarding research translation and impact assessment

The anticipated outcomes will be greater translation of research within CRE-Stroke and an evidence-based report of the impact of CRE-Stroke. The setting will be CRE-Stroke, which brings together an interdisciplinary team of internationally recognised and aspiring researchers primarily from the two major stroke research centres in Australia, namely the Florey Institute of Neuroscience and Mental Health in Melbourne, Victoria, and Hunter Medical Research Institute in Newcastle, New South Wales. Associate researchers and affiliates are based at other sites in Australia. This collaborative effort, with close to 300 network members, is worth AUD 2.5 million over 5 years and unifies stroke researchers from basic science, rehabilitation, health services research and implementation science and provides opportunities for collaboration with clinicians, policy-makers, industry, consumers and carers.

Participants for this study will be a mix of experienced, early career and student researchers associated with all research streams within CRE-Stroke and stakeholders such as clinicians, policy-makers, representatives of consumer organisations, advocates, and stroke carers and survivors.

The framework will be applied to all five research streams of CRE-Stroke (described in Table 1). The aims are to provide transparency to the translation process, increase capacity to improve the speed of translation (when applied prospectively) and, ultimately, to promote a systematic assessment of the impact of these research streams.

**Table 1:** Centre for Research Excellence in Stroke Rehabilitation and Brain Recovery (CRE-Stroke) research streams used for implementation of the Framework to Assess the Impact of Translational health research (FAIT)

Research stream	Synopsis
Basic science	Persistent psychological distress and fatigue are common problems affecting stroke survivor rehabilitation. These problems are under-researched because they are difficult to assess with existing techniques. The Basic Science workstream will address this need through two basic science projects – the first will assess chronic stress in recovering stroke survivors through the measurement of hair cortisol. The second will explore the relationship between inflammation and post-stroke recovery.
Neuroimaging	This workstream addresses two broad themes, namely (1) early (post-stroke) neuroimaging to predict stroke recovery and develop neuroimaging and clinical profiles to inform potential for recovery and allow better stratification of stroke patients in clinical trials, and (2) serial neuroimaging as a biological marker of stroke recovery and ability to benefit from rehabilitation and to inform targeted approaches to rehabilitation interventions. The aim is to learn the characteristics of brain function, structure and metabolism associated with recovery and rehabilitation in those who recover well. This will help inform individual tailoring of rehabilitation interventional approaches.
Clinical trials	This workstream will facilitate the following outcomes with regard to clinical trials in stroke rehabilitation science: improved quality, greater standardisation and increased trial size. Specifically, the workstream will: (1) Develop a platform for rehabilitation trials, with sharing of procedures and resources to increase efficiencies (2) Develop a national training programme (3) Pool trial data to allow novel hypotheses to be developed and improve patient stratification. It is expected that new trials using this information will be developed.
Implementation science	This workstream aims to maximise the translation of effective and cost-effective research from the CRE-Stroke into real-world use and contribute new knowledge to the field of implementation science. The workstream will develop an education programme, a research implementation template, and implementation models and strategies (products) to transfer knowledge from researchers to end-users. This work will be underpinned by appropriate theoretical frameworks to understand the factors that impede or enable implementation to occur at a patient, clinical and/or system level. The effectiveness of implementation models and strategies will be measured and reported.
Data linkage	This workstream will (1) work to link databases in order to better understand the full survivor journey in hospitals and show the associations between the type and quality of care received with longer-term outcomes; (2) facilitate access to already linked datasets held by CRE-Stroke investigators; (3) provide a high level Standard Operating Procedure to facilitate access to available or newly established linked datasets related to stroke rehabilitation; and (4) create new knowledge through the analysis of linked data. This workstream does not intend to be the custodian of linked datasets. However, it will provide a universal governance process for using linked data of relevance to stroke rehabilitation.

The study design involves a four-stage sequential mixed method design, summarised as follows:

- **Phase 1:** Develop a modified programme logic model for each of CRE-Stroke’s five research streams. A scoping review will be used to identify potential benefits from the work of CRE-Stroke and values or sources of value associated with those benefits.
- **Phase 2:** The implementation of FAIT focusing on data collection of evidence to indicate the achievement of process, outcome and impact metrics. This phase will also incorporate a process evaluation to collect participants’ perceptions of FAIT and its implementation.
- **Phase 3:** The package of FAIT methodologies for impact assessment, namely quantified metrics [28], economic assessment and narratives, will be used to assess the impact of the five research streams.
- **Phase 4:** The results will be summarised and presented by way of scorecards, including narratives describing the process by which the CRE-Stroke research translated and generated impact. The outcomes of both the implementation of FAIT and the results of the impact assessment of the five research streams will be compiled and disseminated.

The following sections provide details about the methods for each of the four phases of the study, which may overlap slightly in practice.

### Phase 1

#### Modified programme logic models

“*A logic model is a systematic and visual way to present and share your understanding of the relationships among the resources you have to operate your program, the activities you plan, and the changes or results you hope to achieve*” [29]. A modified programme logic model underpins all the three FAIT impact assessment methods. There are two modifications for FAIT – the main modification relates to the insertion of ‘end users’, with the advantage of identifying who will use the research outputs for impact assessment purposes. In the context of FAIT, end users are defined as collaborators along the pathway to impact that are co-creators and/or co-users of the research outputs [27]. Within CRE-Stroke, this includes clinicians, rehabilitation providers, consumer representatives, industry partners, stroke survivors and carers. Another modification is the introduction of process and output metrics in addition to impact metrics to provide greater transparency between the aims and intended impacts of the research. Process metrics help identify when key activities of the research have occurred. Output metrics help identify when key outputs or products of the research have been produced. Impact metrics reflect the consequence of the research output being used by end users.

**Fig. 1:**
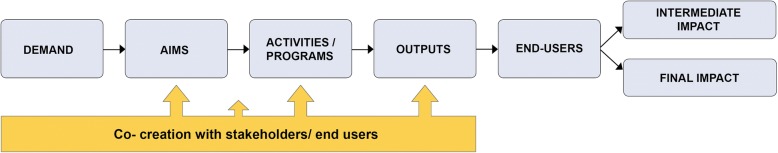
Modified programme logic model

The purpose of the logic models within CRE-Stroke will be to provide strategic maps of how each of the five research streams plans to generate impact and link the aims of each stream to the research activities. These activities should produce an output that, when utilised by an end user, creates an opportunity for the generation of impact. While recognising that translation is a multidirectional phenomenon, this approach provides ‘line of sight’ from need to research to impact, outlined in Fig. 1.

The value in articulating these processes in a programme logic model is to give transparency to how researchers anticipate their project will generate impact, including any interim impacts. It also provides an opportunity to include activities that have (in the literature) been associated with successful translation and the generation of impact such as engagement with end users upfront [30]. Further, logic models also provide a framework for the development of metrics and the collection of evidence of achievement of translational activities and impact.

Five modified programme logic models have been developed collaboratively with CRE-Stroke researchers during a planning workshop for CRE-Stroke. While programme logic models appear linear within Fig. 1 (necessary for the development of a logic model) their application, including project development and refinement, is, in most part, non-linear and iterative in nature. Hence, the logic models are intended to be living documents open to change at all stages of the research pipeline to ensure they capture the actual translational pathways to impact over the lifecycle of CRE-Stroke.

#### Scoping review

A scoping review will be used to identify indicators of impact that will inform the benefits that may be expected from the work of CRE-Stroke, as guided by its objectives. For example, the Becker Medical Library Model for Assessment of Research Impact consists of a list of indicators to document evidence of biomedical research impact [31]. To avoid unnecessary duplication, existing indicators such as these will be the starting point for selection of impact metrics and supplemented with more customised metrics specific to each research stream. These existing indicators will also be used to identify potential values or sources of value associated with those anticipated benefits.

The review process will follow the Joanna Briggs Institute guideline for scoping reviews [32]. While still methodical, scoping reviews are typically broader in their focus with less restrictive inclusion criteria than systematic reviews [33]. The review will be used to map the key concepts underpinning the assessment of impact on the delivery of stroke rehabilitation research. As outlined in the Joanna Briggs Institute guideline, a three-step search strategy will be used. Provisionally, step 1 will involve the development of a literature search strategy including appropriate MeSH and free-text terms that can be used to source articles from a subset of relevant databases. CRE-Stroke researchers will be consulted during this phase of the review. Step 2 will involve screening of the title and abstract of any retrieved papers and of the index terms used to describe the articles. A second search will then be undertaken using all identified keywords and index terms across all included databases. In step 3, the reference list of all identified reports and articles will be hand-searched for additional studies. In this review, literature will be drawn from both economic (e.g. Econlit and Jstore) and general health and medical academic databases (e.g. Medline, Embase, CINAHL, Cochrane Database of Systematic Reviews). The searches will also extend to Google Scholar and Google to identify grey literature from government departments, international organisations and research funders. The searches will be limited to articles published in English between 1995 and 2017. This timeframe is considered appropriate because knowledge translation, a precursor to impact assessment, gained prominence from the late 1990s [34].

The data from the review will be charted to record categories of impact and key benefits that may be anticipated from CRE-Stroke. In line with recommended scoping review guidelines, the charting of results will be iterative [33, 35]. No formal assessment of the quality of the studies will be undertaken, consistent with the methodology for a scoping review.

### Phase 2

#### Monitoring, feedback and data collection for process evaluation

Phase 2 of the study will comprise monitoring progress of all research streams over remaining months of CRE-Stroke operations until end December 2019. This will entail sharing the programme logic models with all CRE-Stroke members and affiliates, allowing for feedback and modifications to the five models at 6-monthly intervals. Through a process of monitoring and feedback, teams from each stream will have the opportunity to assess how they are tracking against their planned activities, outputs and intended impacts, to provide evidence of achievement of process, output and impact goals, and to refine their research translation and engagement activities to maximise impact. In addition, CRE-Stroke members and affiliates will be exposed to current thinking around research translation, implementation and impact through workshops, webinars and forums conducted by CRE-Stroke.

Data collection for this stage of the study will also involve a series of online and telephone surveys of CRE-Stroke researchers and associates to elicit their perceptions of FAIT, determine if the framework encourages translational behaviours and how the implementation of the framework can be improved. This is in line with a process evaluation of the application of FAIT. Participants will also be asked to articulate which aspects of the framework are effective and which need refinement.

### Phase 3

#### Analysis of collected data – research impact assessment and valuation

**Fig. 2:**
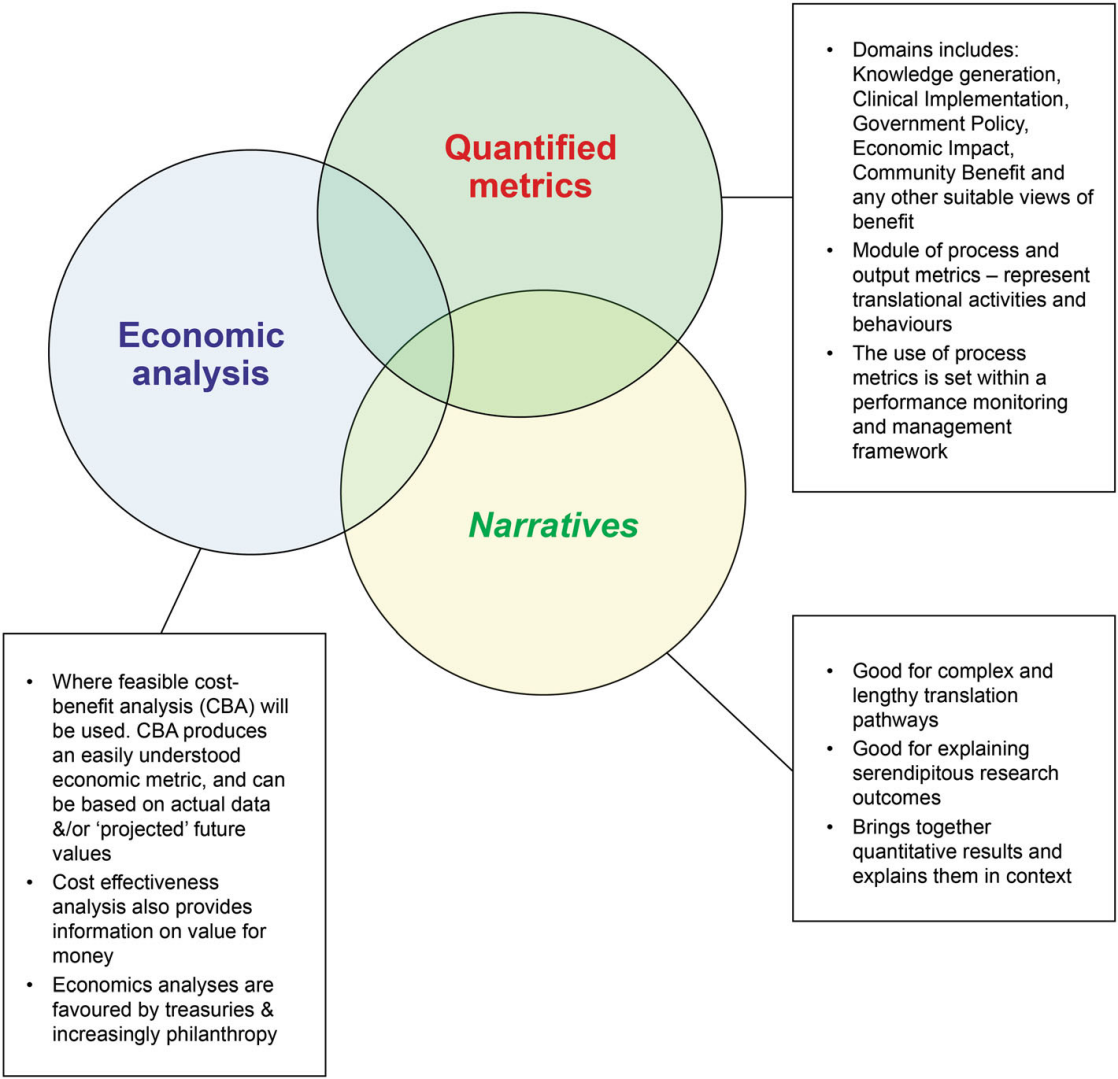
Validated impact assessment methodologies used in FAIT

Currently, there is no single assessment method capable of capturing the impacts stemming from health and medical research. Therefore, FAIT employs a combination of three separate but integrated proven methods, namely quantified metrics [36], economic assessment [37] and narratives of the process by which the research in question translates and generates impact (Fig. 2). Using qualitative project examples, the narratives will be triangulated against the quantified metrics and the economic assessment to validate the impact of the research in question.

#### Impact metrics for the modified Payback model

The impact metrics referred to in FAIT are a variation of the methods used in the Payback Framework [38]. The Payback Framework was originally developed to examine the ‘impact’ or ‘payback’ of health services research. It consists of a logic model representation of the complete research process and a series of categories to classify paybacks from research from the more traditional academic benefits of knowledge production to wider benefits to society. Impact metrics will be organised under broad domains of benefit such as impacts on knowledge, policy, practice, economics and community. These impact metrics will be structured to support the planned economic assessment. Robust impact metrics that are contextually relevant to stroke rehabilitation research, such as inclusion in stroke rehabilitation guidelines or improvement in arm function post-stroke, will be selected with consideration to objectivity, administrative efficiency, transparency and comparability, as well as their ability to be verified. Additionally, some of these impact metrics are more like indicators that can be evidenced rather than measured (e.g. inclusion in guidelines) and will be reported accordingly.

#### Economic assessment

The economic assessment will involve a comparison of the costs associated with developing and implementing the five research streams versus a calculated value for the expected impact or benefit of the funded research. This will provide an estimate of return on investment (ROI). The nature of some CRE-Stroke activities and available data on costs may impact on the type of economic assessment that is feasible and useful. The planned assessment will collect, on a case-by-case basis, the resources used to fund the research, including non-CRE-Stroke funding expended on each activity. The cost of running the CRE-Stroke programme will be appropriately apportioned across the five research streams, including any cross-cutting programmes. Additional costs in utilising the research outputs of each stream will also be included. For example, the development of serial neuroimaging as a biological marker of stroke recovery will help inform individual tailoring of rehabilitation interventional approaches. Resources will be consumed to develop and evaluate the protocols and information required to conduct such imaging. Implementation of that protocol might increase the number of imaging tests performed on patients with stroke. These tests may incur additional costs to the health system and their costs will be accounted for. Implementation of that imaging protocol might also have positive impacts on short- and long-term recovery from stroke, which can be reported as downstream savings to the health system and society.

The programme logic models will assist in articulating programme inputs, expected outputs, uptake and ultimate impact. The total calculated expected costs and benefits will be combined to estimate ROI. Depending on the focus and stage of each research stream, three broad steps will be involved in the economic assessment, namely (1) identification and assessment of resource use, (2) assessment and valuation of the expected impact, where possible, and (3) comparison of the costs and expected impacts, where possible, in a single metric. Where practical, the analysis will assume a societal perspective to ensure all possible costs and benefits are accounted for. The time horizon for the assessment will be bounded in the base case analysis by the period during which the programme received core funding, i.e. 2015–2019. Expected costs and impacts will be reported in net present value terms and streams of projected future costs and benefits will be discounted at a base case rate of 3% [39].

- Identification, assessment and valuation of resource use

Resource use associated with development and delivery of the research/interventions will be costed using financial and administrative records from the respective research teams. The costs associated with translation of the project findings and outputs will include any costs (including opportunity costs) incurred by the various health service organisations such as costs related to practice change. As stated above, it may be problematic to collect data to inform these costs. However, some attempt will be made to model these costs using administration records and detailed descriptions of uptake obtained from health services.

- Assessment and valuation of the expected impact

Impact will be calculated for selected domains for each of the five programme logic models. The calculations will be adjusted for risk to give the expected value of the impact. Attribution will be assigned at a conservative rate, the value of which will be informed by administrative and evaluation records and qualified during researcher and other interviews. Projected valuations will include a ‘drop-off’ factor to account for waning benefit over time [39]. Variation in both attribution and drop off factors will be included in a sensitivity analysis. Any and all assumptions underpinning the analysis will be made explicit in the reporting of the results.

#### Narratives (case studies)

The FAIT approach also incorporates the use of illustrative examples or narratives that will be compiled for each research stream to describe, in more qualitative terms, how translation occurred and how impact was generated for each stream [27]. The use of narratives has been the basis of the research evaluation system currently used in the United Kingdom [40]. Narratives are useful for describing the often complex pathways for research translation and can be powerful tools for communicating the nature and extent of research translation and, ultimately, research impact with policy-makers, funders and the wider community. They also enable quantitative findings to be placed in context and are an opportunity to explain variances in research costs, outputs and impacts. In this application within CRE-Stroke, it is expected that the narratives will be supported with quantitative evidence extracted from the quantified metrics and economic assessments and will be used to triangulate and validate the impact findings. It is expected that the narratives will be informed by interviews with key CRE-Stroke researchers, affiliates and key stakeholders, including end users of the research such as clinicians and health service staff. This collaborative and prospective approach to the development of the narratives will render them less likely to be impacted by biases such as selective memory, which often characterise narratives based only on self-reports [41].

### Phase 4

#### Reporting and recommendations around the implementation of FAIT

**Fig. 3:**
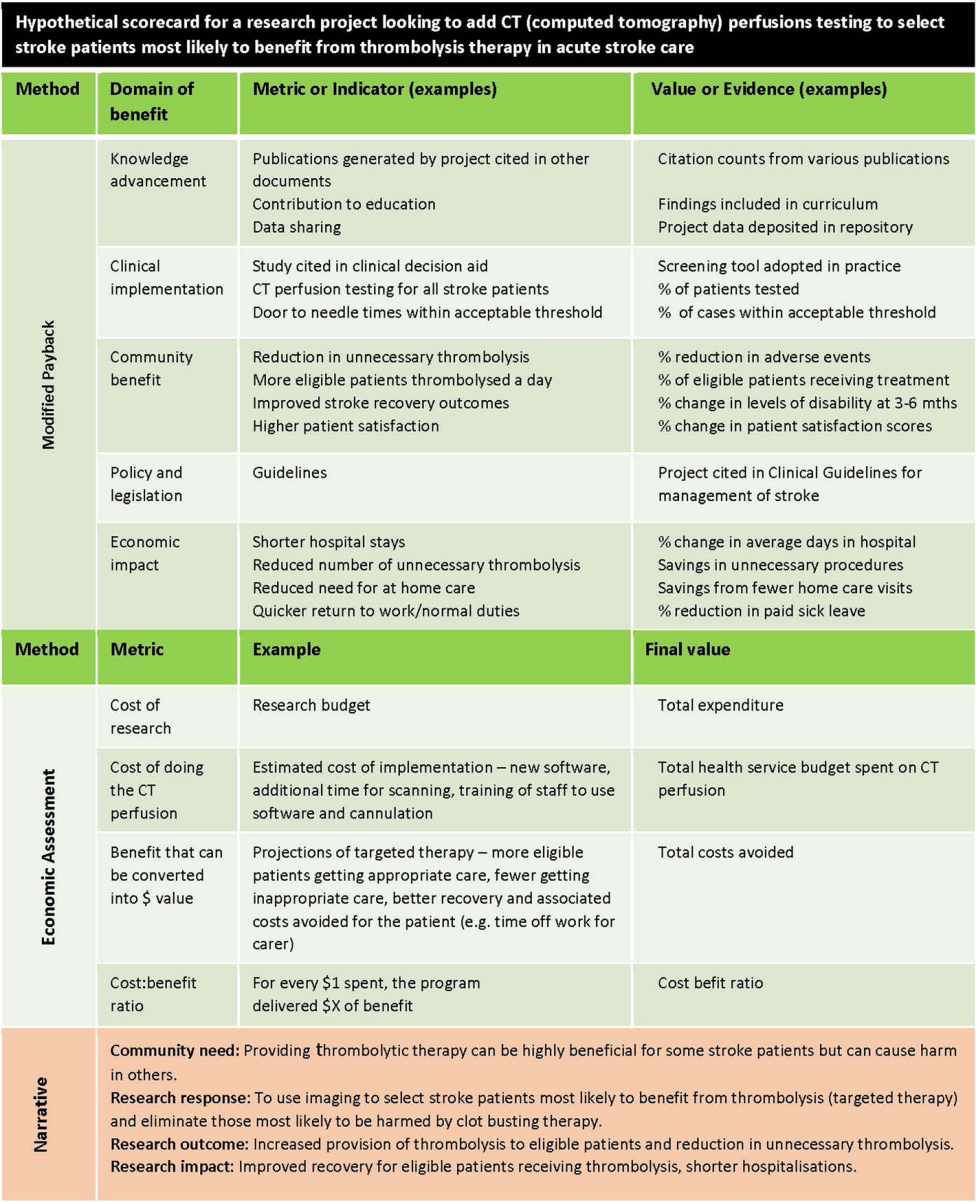
Hypothetical scorecard for reporting Framework to Assess the Impact of Translational health research (FAIT) impact findings

Outcome 1: The results, including the narratives, will be summarised and reported by way of a scorecard (see Fig. 3 for a hypothetical scorecard). These scorecards will form the basis of CRE-Stroke reporting of the translation and impact of its five research streams and potentially dovetail into an overall evaluation of the CRE.

Outcome 2: The findings from the implementation of FAIT within CRE-Stroke and specifically about its applicability to research streams, as opposed to research projects, will be compiled. A workshop with key CRE-Stroke researchers and stakeholders will be conducted to discuss the findings and obtain feedback with a view to refining FAIT for future use.

### Limitations

This study is being conducted in a real-world setting. Impact assessments are resource intensive and, although the prospective collection of evidence is more cost-effective, not all the required data can be collected prospectively. Final metrics for the Payback assessment and data for the narratives and economic assessments for each stream will be based on what can feasibly be collected versus an ideal list of impact metrics. The lag between research translation and impact means that valuations may need to be undertaken with reference to interim rather than final impacts. For CRE-Stroke streams that are more advanced, this constraint will be less problematic compared to projects that have commenced more recently. Conduct of the study in a real-world setting means there are no controls (counterfactuals or what would have happened in the absence of CRE-Stroke), thus attribution of impact for all five streams will be necessarily conservative. They will also be constrained, in some cases, by the evidence available to substantiate claims that specific impacts are attributable to the research being assessed. Finally, FAIT is project based and is being applied to five research streams. A limitation, therefore, is that this study will not assess the impact of individual CRE-Stroke research projects, including randomised controlled trials that could potentially have large impacts once fully translated.

## Discussion

This protocol describes a mixed methods study to apply a systematic framework to encourage research translation and assess impact beyond academic outputs. Academic outputs are already well reported by Excellence in Research for Australia, however, they provide an incomplete assessment of ROI, particularly for health and medical research. Most existing impact assessment frameworks are retrospective and provide accountability for past research investments. FAIT is prospective in design and incorporates monitoring and feedback with the explicit aim of enhancing translation and impact. This study will capture processes, outputs and impacts generated across the spectrum of stroke rehabilitation research from discovery to applied science, utilise cost-effective data collection techniques and facilitate a wider range of reportable outputs and impact of research for CRE-Stroke. It stands to make a solid contribution to our understanding of research translation and impact assessment from the perspective of a programme of research that encourages interdisciplinary capacity-building.

## Abbreviations

AUD: Australian Dollar
CRE-Stroke: Centre for Research Excellence in Stroke Rehabilitation and Brain Recovery
FAIT: Framework to Assess the Impact from Translational health research
NHMRC: National Health and Medical Research Council
ROI: Return on investment
